# Aptamer-Targeted Drug Delivery for *Staphylococcus aureus* Biofilm

**DOI:** 10.3389/fcimb.2022.814340

**Published:** 2022-04-29

**Authors:** Pernille Ommen, Line Hansen, Bente K. Hansen, Hieu Vu-Quang, Jørgen Kjems, Rikke L. Meyer

**Affiliations:** ^1^Interdisciplinary Nanoscience Center, Aarhus University, Aarhus C, Denmark; ^2^Department of Molecular Biology and Genetics, Aarhus University, Aarhus C, Denmark; ^3^Department of Bioscience, Aarhus University, Aarhus C, Denmark

**Keywords:** aptamer, *Staphylococcus aureus*, biofilm, drug delivery, liposome, rifampicin, vancomycin

## Abstract

Treatment of *Staphylococcus aureus* biofilm infections using conventional antibiotic therapy is challenging as only doses that are sublethal to the biofilm can be administered safely to patients. A potential solution to this challenge is targeted drug delivery. In this study, we tailored an aptamer-targeted liposomal drug delivery system for accumulation and delivery of antibiotics locally in *S. aureus* biofilm. In our search for a suitable targeting ligand, we identified six DNA aptamers that bound to *S. aureus* cells in biofilms, and we demonstrated that one of these aptamers could facilitate accumulation of liposomes around *S. aureus* cells inside the biofilm. Aptamer-targeted liposomes encapsulating a combination of vancomycin and rifampicin were able to eradicate *S. aureus* biofilm upon 24 h of treatment *in vitro*. Our results point to that aptamer-targeted drug delivery of antibiotics is a potential new strategy for treatment of *S. aureus* biofilm infections.

## 1 Introduction

A major challenge in infection microbiology is the ineffectiveness of antibiotics against infections caused by bacterial biofilms (Bjarnsholt, 2013). *Staphylococcus aureus* is a biofilm-forming bacterium that is notorious for causing a broad range of persistent tissue- and implant-associated infections (Bhattacharya et al., 2015). The eradication of these biofilm infections is complicated because the bacteria in biofilms are encased in a self-produced extracellular matrix composed of proteins, polysaccharides, and extracellular DNA, which protects them against the host immune system and antimicrobial agents. Moreover, bacteria in biofilms may enter a low metabolic state, which increases their tolerance to antibiotics dramatically (Otto, 2008). As a result, the bacteria in biofilms may tolerate up to 1,000 times higher concentrations of antibiotics than their planktonic counterparts (Høiby et al., 2010). This raises the problem: antibiotics cannot be dosed in a concentration sufficient to eradicate the biofilm without also causing detrimental side effects to the patient. The only recourse is surgical removal of the biofilm; however, this procedure is associated with high costs, and in some cases it is not feasible (Forier et al., 2014; Høiby et al., 2015). Thus, an improved treatment strategy for eradication of biofilm-infections is highly needed.

Targeted drug delivery using nanocarriers is a promising strategy to combat biofilm infections. Targeted delivery ensures release of antibiotics in close proximity to the biofilm, enabling a high local antibiotic concentration with minimal risk to the patient (Forier et al., 2014). For this purpose, liposomes are promising as nanocarriers, and many drug–liposome systems are in clinical use for cancer treatment. Several liposome-based drug delivery systems involving antibiotics have also reached the clinic, including treatment of fungal biofilms (Bulbake et al., 2017). For the treatment of bacterial infections, antibiotics formulated in liposomes have primarily been developed for inhalation to treat respiratory biofilm infections, e.g., in cystic fibrosis patients. Liposome-encapsulated ciprofloxacin Lipoquin™ and Pulmaquin™ are tested in clinical trials, and liposome-encapsulated amikacin Arikayce^®^ has obtained FDA approval for treatment of *Mycobacterium avium complex* (MAC) lung disease (Bassetti et al., 2020). Liposomes are suitable for this kind of drug delivery due to their biocompatibility and ability to carry one or more kinds of therapeutic molecules of both hydrophilic and hydrophobic nature (Schwendener and Schott, 2010; Eloy et al., 2014). They may furthermore improve the pharmacokinetics and reduce the off-target toxicity of the drugs that they carry (Drulis-Kawa and Dorotkiewicz-Jach, 2010; Bulbake et al., 2017). Moreover, encapsulation of antibiotics into liposomes has been shown to improve their efficacy. For example, Sande et al. showed improved killing of methicillin-resistant *S. aureus* by liposomal vancomycin compared to free vancomycin *in vitro* and *in vivo* (Sande et al., 2012). Lastly, liposomes can be targeted to accumulate at a certain site and functionally modified to release their content on demand using internal stimuli, such as pH, or external stimuli such as hyperthermia (Bibi et al., 2012).

In order for the targeted drug delivery strategy to be successful, it is key to finding a targeting agent that will provide a specific interaction with the bacteria, ensuring accumulation and release of antibiotics inside the biofilm. For *S. aureus*, the recognition ligand may either be a surface protein, such as staphylococcal protein A (Meeker et al., 2016), or a cell wall component (Meng et al., 2016). As targeting agent, aptamers are uniquely suited. These single-stranded oligonucleotides fold into a three-dimensional structure and bind their target specifically with high affinity by structural recognition (Sun and Zu, 2015). Several aptamers specifically recognizing free-living *S. aureus* cells have already been developed (Cao et al., 2009; Chang et al., 2013; Moon et al., 2015; Stoltenburg et al., 2015). Among these aptamers, some have shown potential as targeting agents for drug delivery to planktonic *S. aureus* (Kavruk et al., 2015). Nevertheless, direct translation for use of these aptamers as targeting agents toward *S. aureus* in biofilms is not given as bacteria in biofilms exert several differences, e.g., altered surface protein expression from those living freely (Resch et al., 2006).

In this study, we aim to identify an aptamer suitable as *S. aureus* biofilm targeting agent. We hypothesize that aptamer-targeted antibiotic-loaded liposomes will bind to the surface of *S. aureus* cells, accumulate in biofilms, and eradicate the biofilm upon release of content. By applying a temperature-sensitive liposomal formulation, it is possible to trigger the release of the content by mild hyperthermia (Needham et al., 2013). The combination of accumulating liposomes at the surface of *S. aureus* and the triggered release of antibiotics from them in a bolus fashion can potentially lead to a high concentration of antibiotics in close proximity to the bacteria. We hypothesize that this combinatorial strategy will eradicate the biofilm more efficiently than a passive release of the content during 24 h of treatment.

We first compared the ability of different *S. aureus-*specific aptamers to target *S. aureus* in biofilms and then selected a promising candidate for use to target and accumulate liposomes in *S. aureus* biofilms. Lastly, we investigated if retained targeted liposomes loaded with vancomycin and rifampicin could eradicate *S. aureus* biofilms both upon and without hyperthermia-induced release. Our results show that aptamers can bind and accumulate antibiotics-loaded liposomes around *S. aureus* cells in biofilms. The retained liposomes eradicated *S. aureus* biofilms during 24 h of treatment; however, an initial hyperthermia-induced release counteracted this effect. Even so, our results point to that aptamer-targeted drug delivery of antibiotics is a promising approach to eradicate *S. aureus* biofilms.

## 2 Methods

### 2.1 Bacterial Strain and Biofilm Growth Conditions

*S. aureus* DSM20231 was used in this study. The bacterial culture was stored at -80°C in 50% glycerol stocks, and prior to experiments 1 μl was subcultured on brain heart infusion (BHI) (Sigma-Aldrich) agar plates overnight at 37°C. Colony plates were kept at 4°C up to 1 month. Bacterial cultures for experiments were then prepared by inoculating a single colony in 25 ml 3.7% BHI broth for 16–20 h at 37°C shaking at 180 rpm.

The biofilms in this study were grown in BHI broth enriched with 5% human plasma. Human plasma was obtained from healthy donor blood samples collected in tubes coated with EDTA (1.8 mg EDTA/ml blood, BD Vacutainer**^®^**, Becton Dickinson, Franklin Lakes, NJ, USA). Plasma was isolated by centrifugation at minimum 2,000 g for 15 min at 5°C. Pools of plasma from multiple donors were then prepared and stored at -20°C or -80°C.

Biofilms were formed in 96-well microtiter plates (Sarstedt 83.3924.500) using the following protocol: prior to inoculation, wells were preconditioned in 100 μl BHI enriched with 50% plasma for 30 min at 37°C. 80 μl pre-conditioning medium was removed before addition of 180 μl overnight culture diluted to OD_600_ = 0.5 (final volume = 200 μl, 5% plasma). Upon 30 min of inoculation at 37°C, 180 μl was replaced with fresh BHI enriched with 5% to remove most of the planktonic bacteria, which ensures better nutrient availability for the adhered bacteria. Biofilms were formed at 37°C for 24 h.

### 2.2 Aptamer Screening

Aptamers were ordered as custom DNA oligos from Integrated DNA Technologies (IDT, Coralville, IA, USA) or Sigma-Aldrich (St. Louis, MO, USA).

*Fluorescence Labeling of Aptamers*. Aptamers were fluorescently labeled by adding 5-propargylamino-ddUTP-Cy5 (Jena Bioscience, Thüringen, Germany) to the 3′ end using the Terminal Deoxynucleotidyl Transferase (TdT) kit (Sigma-Aldrich). For labeling of 1 nmol aptamer, the following optimized protocol was used: 2 μl aptamer (0.5 mM) was mixed with 4 μl 5× TdT buffer, 4 μl CoCl_2_ (25 mM), 4 μl propargylamino-ddUTP-Cy5 (0.5 mM), 2 μl TdT, and 4 μl nuclease-free water for a total volume of 20 μl. The reaction was incubated at 37°C for 1 h. DNA was recovered by ethanol precipitation, redissolved in binding buffer (**Supplementary Table S1**), and purified using Illustra™ MicroSpin™ G-50 columns for removal of excess Cy5-ddUTP and cobalt.

To confirm successful labeling of the aptamer, a 0.25-pmol sample from each reaction was loaded in 10% denaturing polyacrylamide gel. Electrophoresis was run in 1× Tris–borate–EDTA (TBE) buffer at 600 V for 1 h. The gel was subsequently stained for nucleic acids with SYBR^®^ Gold (Thermo Fisher Scientific, Waltham, MA, USA). Images for analysis were acquired using the GE Healthcare Typhoon Trio Variable Mode Imager System (GE Healthcare Life Sciences, Chicago, IL, USA), which enables quantification of the fluorescence emitted from, e.g., gels or microwell plates. ImageQuant TL software (GE Healthcare Life Sciences) was used for image analyses.

*Aptamer Binding to S. aureus Biofilms.* Biofilms were grown as described in 2.1. Fluorescently labeled aptamers were diluted in binding buffer to a concentration of 250 nM and folded as described in **Supplementary Table S1**. 50 μl aptamer solution was added to each biofilm after rinsing the biofilm once in 200 μl PBS and twice in 200 μl binding buffer. Aptamers were incubated with biofilms at 37°C for 1 h. Unbound material was then removed by washing biofilms three times in binding buffer. Binding of fluorescently labeled aptamers in biofilms was imaged using the GE Healthcare Typhoon Trio Variable Mode Imager System. Each biofilm was then stained in 100 μl 5 μM SYTO9 (Thermo Fisher Scientific) and imaged again using the Typhoon Trio variable-mode imager system. ImageQuant TL software was used for quantification of Cy5 and SYTO9 signal. The experiment was performed in triplicate.

For a detailed visualization of aptamer binding in biofilms, the Zeiss LSM700 confocal laser scanning microscope (CLSM) was used. For this purpose, biofilms were prepared in microtiter plates for microscopy (ibidi, Gräfelfing, Germany, #89621). Biofilms were stained with 100 μl 5 μM SYTO9. Images were acquired using a ×63 NA1.4 Plan Apochromat Oil Immersion Objective (Zeiss) and excitation with lasers 488 and 639 nm.

### 2.3 Aptamer Stability

Aptamer stability in human plasma was investigated by mixing 5 pmol aptamer with 40 μl human plasma. This human plasma was obtained from healthy donor blood samples collected in tubes coated with lithium heparin (170 I.U. heparin/ml blood, BD Vacutainer**^®^**, Becton Dickinson). The plasma was isolated as described in Section 2.1. The mixture was incubated at 37°C, and at 0, 30, 60, and 120 min and 16 h samples were withdrawn. The degradation of aptamers was investigated by loading 0.125 pmol of each sample in 10% denaturing polyacrylamide gel. Electrophoresis was run and analyzed as described in Section 2.2.

### 2.4 Preparation of Liposomes

Low-temperature sensitive liposomes (Needham et al., 2013) composed of 1,2-dipalmitoyl-sn-glycero-3-phosphocholine (DPPC), 1-stearoyl-2-hydroxy-sn-glycero-3-phosphocholine (MSPC), and 1,2-distearoyl-sn-glycero-3-phosphoethanolamine-N-[dibenzocyclooctyl(polyethylene glycol)-2000] (ammonium salt) (DSPE-PEG2000-DBCO) (Avanti Polar Lipids, Alabaster, AL, USA) and 1,2-distearoyl-sn-glycero-3-phosphoethanolamine-n-[methoxy(polyethylene glycol)-2000] (DSPE-PEG2000) (Laysan Bio Inc., Arab, AL, USA) were prepared using the thin lipid film hydration method followed by extrusion through polycarbonate membrane filters (Zhang, 2017). Briefly, the lipids were dissolved in a mixture of chloroform and methanol (4:1 v/v) and then mixed in a round-bottomed glass flask in molar ratio DPPC: MSPC: DSPE-PEG(2000) 86.5: 9.7: 3.8. Lastly, chloroform was added for a lipid concentration of 10 mg/ml. The flask was placed in a 45°C water bath and connected to a vacuum rotary evaporation system to remove the organic solvent and form a thin lipid film on the inside surface of the flask. The thin lipid film was dried overnight under vacuum to remove any residual solvent.

*Fluorescent Dye-Loaded Liposomes.* The lipid film was hydrated in PBS containing 1 μM MSPC and either 30 μg/ml sulforhodamine B (Sigma-Aldrich) or 70 mM calcein (Sigma-Aldrich) by gently stirring the tube in a 50°C water bath for 10 min. The sample was extruded through 800-, 400-, 200-, and then 100-nm polycarbonate membrane filters (Whatman^®^ Nuclepore Track-Etched Membranes, Sigma-Aldrich) (11 times each) with a syringe extrusion device (Avanti Mini Extruder, Avanti Polar Lipids) kept at 50°C. Unincorporated sulforhodamine B was removed using Micro Bio-Spin Column with Bio-Gel P30 (Bio-Rad, Hercules, CA, USA). Unincorporated calcein was removed using Amicon Ultra-15 Centrifugal Filter Units (100K) (Merck). Liposomes were stored at 4°C.

*Vancomycin- and Rifampicin-Loaded Liposomes.* Rifampicin (0.05 mg) (Sigma-Aldrich) was dissolved together with the lipids (35 μmol) in the organic solvent, and a lipid film was prepared as described above. The thin lipid film was hydrated in ddH_2_O containing 1 μM MSPC by gently stirring the tube in a 50°C water bath for 10 min. The sample was then extruded through 800-, 400-, 200-, and 100-nm polycarbonate membrane filters (11 times each) as described above. Vancomycin was encapsulated using the dehydration–rehydration method (Antimisiaris, 2010). Briefly, 40 mg vancomycin (Hospira, Lake Forest, IL, USA) dissolved in 2 ml 1/10 PBS diluted in ddH_2_O was mixed with 1 ml liposome solution. Sucrose was then added in a 1:1 liposome: sucrose ratio (weight/weight) to protect the liposomes during freeze-drying. The solution was freeze-dried overnight under vacuum. The powder was rehydrated in 200 μl ddH_2_O (1/10 volume of vancomycin solution) preheated to 50°C. The solution was kept at 50°C for 30 min. 1.8 ml preheated PBS was added, and the liposome solution was incubated at 50°C for another 30 min. The liposome solution was then cooled to 4°C. Liposomes were purified from unincorporated rifampicin and unencapsulated vancomycin using a PD-10 column (GE Healthcare) and Amicon Ultra-15 Centrifugal Filter Units (100K). Liposomes were stored at 4°C.

### 2.5 Conjugation of Aptamers to Liposomes

Liposomes were functionalized with aptamers using copper-free click chemistry by addition of N_3_-labeled aptamers to DBCO-functionalized liposomes. Liposomes were functionalized with DBCO by replacing 0.5-1 molar % of DSPE-PEG(2000) with DSPE-PEG(2000)-DBCO in the liposome preparation. Aptamers were 3′-end-labeled with a ddUTP-PEG(8)-N_3_ or ddUTP-PEG(24)-N_3_ using TdT as described in Section 2.2. Prior to conjugation, N_3_-modified aptamers were folded as described in **Supplementary Table S1**. Folded N_3_-modified aptamers were added in DBCO:aptamer ratios of 1:0, 1:1, 2:1, and 4:1. The conjugation reaction was run for 20–22 h in PBS in a reaction volume of 35 μl. To minimize leak of antibiotics, the reaction was run at 4°C for antibiotics-loaded liposome. Otherwise, the reaction was run at 25°C.

Non-conjugated aptamers were removed by filtration using Amicon**^®^** Ultra 100K centrifugal filters (Merck, Darmstadt, Germany). Aptamer functionalized liposomes were stored at 4°C until use.

### 2.6 Characterization of Aptamer-Functionalized Liposomes

*Agarose Gel Electrophoresis*. To confirm successful conjugation of aptamers to the surface of the liposomes, a non-purified sample from each reaction was loaded into a 1% agarose gel supplemented with 10 μl SYBR™ Safe DNA Gel Stain (Thermo Fisher Scientific). Electrophoresis was run in 1× TBE buffer at 120 V for 60 min. Images for analysis were acquired using the Bio-Rad Gel Doc EZ Imager.

*Dynamic Light Scattering (DLS)*. The mean diameter and particle size distribution of the liposomes were measured by dynamic light scattering (DLS) using Zetasizer Nano ZS (Malvern Instruments, Malvern, UK).

*Transmission Electron Microscopy (TEM)*. TEM was used for investigating liposome morphology. Samples were negatively stained with 1% phosphotungstic acid and visualized in a FEI Tecnai G2 Spirit (Bio)twin transmission electron microscope.

### 2.7 Interaction of Aptamer-Functionalized Sulforhodamine B-Loaded Liposomes With *S. aureus* Biofilms

Twenty-four-hour-old biofilms were prepared as described in Section 2.1, rinsed three times in binding buffer (PBS), and 50 μl of binding buffer containing rhodamine B-loaded liposomes functionalized with different amounts of aptamer SA31 or SCRAM (DBCO:apt ratios of 1:0, 1:1, 2:1, and 4:1) was added. The liposomes were allowed to bind for 1 h at 37°C. Biofilms were then washed five times in PBS. The amount of bound liposomes in the biofilm was imaged using the Typhoon Trio+ variable mode imager and quantified using ImageQuant TL software. To visualize penetration and binding in detail, the Zeiss LSM700 confocal laser scanning microscope was used. For this analysis, biofilms were stained in 100 μl 10 μM SYTO41 (Thermo Fisher Scientific). Images were acquired using lasers 405 and 555 nm for excitation and a ×100 NA1.4 Plan Apochromat Oil Immersion Objective (Zeiss, Jena, Germany).

### 2.8 Characterization of Temperature-Induced Release From Liposomes

The temperature-sensitive liposome formulation used in this study had a melting temperature of approximately 42°C (Needham et al., 2013), at which a release of content is triggered. To investigate the release profile in detail, the liposomes were incubated in a 96-well microtiter plate submerged in a water bath. The water bath was kept at 45°C to ensure that at least 42°C was reached in the wells. Previous optimization studies (data not shown) confirmed that this temperature was necessary in order to reach the melting temperature of our liposome formulation. The temperature was monitored in a well containing 200 μl PBS using an EasyLog USB temperature probe (Lascar Electronics, Erie, PA, USA).

*Release of Model Drug Calcein*. Calcein-loaded liposomes were diluted in PBS (1:10) and incubated for 0, 5, 10, and 15 min before withdrawal of the sample. The samples were kept on ice until analysis to stop the release of calcein. 80 μl of each sample was transferred to black 96-well microtiter plates (Greiner, Kremsmünster, Austria). The fluorescence was measured using FLUOstar OPTIMA at 520 nm with excitation at 485 nm. The liposomes were then lysed by addition of 10 μl of 10% Triton X-100 (Sigma-Aldrich). Upon 15 min at RT, the fluorescence was measured again using FLUOstar OPTIMA at 520 nm with excitation at 485 nm. The percentage release of calcein was calculated by the following:


$$\%Release=\frac{F_{t}-F_{i}}{F_{f}-F_{i}}*100$$

where F_t_ is the fluorescence at t min, F_i_ is the fluorescence at 0 min, and F_f_ is the total fluorescence after addition of Triton X-100.

*Release of Vancomycin.* Vancomycin-loaded liposomes were diluted in PBS (1:10) and incubated for 0 or 15 min before withdrawal of the sample. A sample of vancomycin-loaded liposomes kept at 37°C for 1 h was included as control. Released vancomycin was separated from liposomes using the 100K Nanosep Centrifugal Device (Pall). The filtrate was diluted 1:2 in ddH_2_O before high-pressure liquid chromatography (HPLC) analysis. The amount of released vancomycin was analyzed by reverse-phase high-pressure liquid chromatography (RP-HPLC) on an Agilent 1200 Series using a Gemini NX C18 column (Phenomenex, Torrance, CA, USA, size: 250 × 4.6 mm) and a 0.1% TFA gradient buffer system (C: 0.1% TFA, B: acetonitrile (MeCN)) with detection at 280 nm. Samples were analyzed using a gradient starting at 100:0 (C:B) reaching 35% MeCN in 2–12 min and then 35%–95% in 3 min, then 95% for 2 min. A flow rate of 0.7 ml/min was used and an injection volume of 50 μl.

A vancomycin standard curve prepared in ddH_2_O ranging from 31.25 to 500 μg/ml was analyzed along with the release samples.

To determine the percentage release, a sample of vancomycin-loaded liposomes was lysed with Triton X-100 (1:1, vol/vol) diluted 1/10 in ddH_2_O, and analyzed along with the release samples.

### 2.9 Killing of *S. aureus* Biofilms by Aptamer-Functionalized Thermosensitive Liposomes Loaded With Vancomycin and Rifampicin

Twenty-four-hour-old biofilms were prepared as described in Section 2.1, rinsed twice in binding buffer (PBS), and 50 μl of binding buffer containing aptamer-functionalized or naked thermosensitive liposomes loaded with vancomycin and rifampicin was added. A total of 1 mg of vancomycin was added to each well. The liposomes were allowed to bind for 1 h at 37°C. Biofilms were then washed five times in 200 μl PBS. Then, 100 μl PBS was added to each well and biofilms were either incubated at 37°C immediately or first heated in a 45°C water bath for 15 min. After 24 h of treatment, biofilms were rinsed in 200 μl PBS. 200 μl PBS was added to each well, and the biofilms were then sonicated for 10 min in a 45-kHz water bath sonicator (USC-T, VWR). The biofilm was then completely dissolved manually by pipetting. Ten-fold dilutions were prepared in PBS from the sonicated suspensions, and 100 μl from each dilution was added to BHI agar plates (detection limit: 10 CFU/ml). The plates were incubated overnight at 37°C and colonies were counted.

### 2.10 Statistical Analyses

All statistical analyses were performed in GraphPad Prism 7.0a (GraphPad Software). The test used in each experiment is noted in the figure captions.

## 3 Results

### 3.1 Identification of Aptamers That Bind *S. aureus* in Biofilms

**Figure 1:**
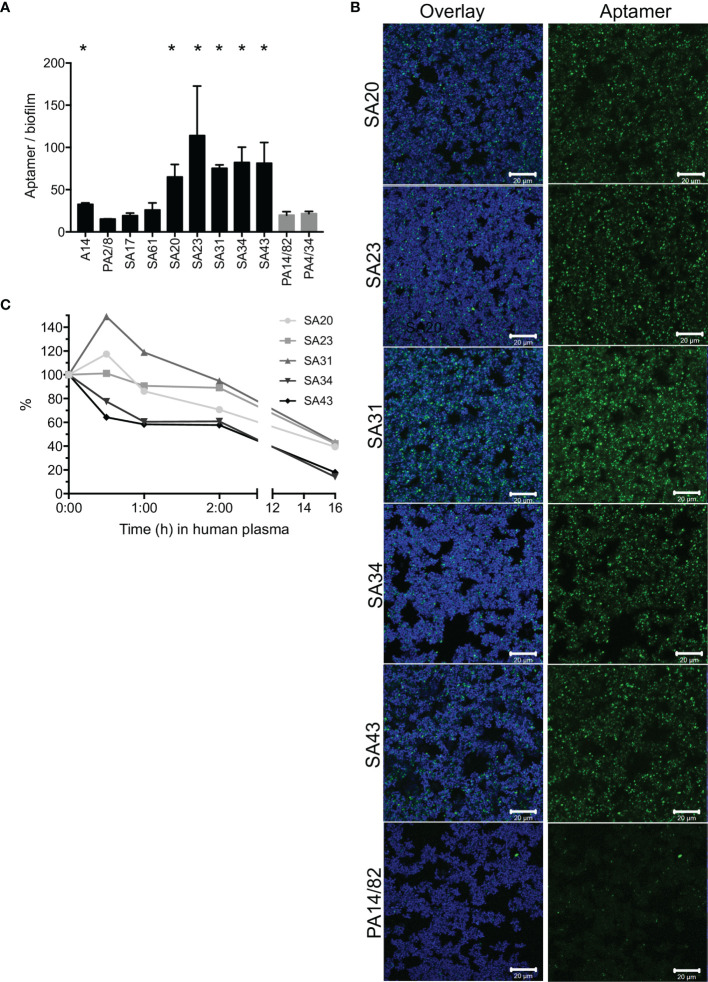
Screening of *S. aureus-*specific DNA aptamers. **(A)** Fluorescence from Cy5-labeled aptamers incubated for 1 h with *S. aureus* biofilms grown in BHI with 5% human plasma. Biofilms were stained with SYTO9, and the aptamer signal was normalized to the SYTO9 signal to account for differences in the amount of biofilm. Graph shows mean +/- SD, n = 3. Binding of *S. aureus*-specific aptamers was compared with the non-specific aptamers PA14/82 and PA4/34; * marks p < 0.05 (unpaired t-test). **(B)** CLSM imaging of Cy5-labeled aptamers (green) to SYTO9-stained *S. aureus* biofilms (blue). **(C)** Stability of SA20-43 in human plasma at 37°C. Samples were withdrawn at the given time points and run in a denaturing gel. The band intensities were quantified and normalized to the intensity at time 0.

To identify an aptamer that would be applicable as a targeting agent, a total of nine aptamers (Cao et al., 2009; Chang et al., 2013; Moon et al., 2015; Stoltenburg et al., 2015) selected toward whole *S. aureus* or *S. aureus* protein A were screened for their binding capacity to *S. aureus* in biofilms. Six aptamers bound significantly better than the non-specific controls, and the signal/noise ratio was particularly high, with five of these (SA20-43, **Figure 1A** and **Supplementary Figure S1**). These biofilm-binding aptamers originated from the same selection toward whole *S. aureus* bacteria (**Supplementary Materials** and **Supplementary Table S1**) (Cao et al., 2009), while, e.g., aptamer PA2/8 selected for binding to Protein A showed no significant binding. CLSM imaging showed that these aptamers co-localized with the bacteria in the biofilm (**Figure 1B**). Furthermore, the aptamers did not only bind to bacteria at the top of the biofilm but penetrated and bound *S. aureus* throughout the entire biofilm (data not shown). Among the five biofilm-binding aptamers imaged, SA31 showed the best binding (**Figure 1B**) and was therefore selected for targeting liposomes to *S. aureus* biofilms. In the initial publication of SA31, its specificity toward two clinical *S. aureus isolates* was confirmed, while it did not bind to clinical isolates of *Staphylococcus epidermidis* or *Streptococcus* (Cao et al., 2009).

Aptamer susceptibility to nuclease degradation is an important consideration when determining the applicability of aptamers as targeting agents. We therefore investigated the degradation of SA20-43 in plasma during 16 h of incubation. SA31 and SA23 appeared more stable than the other aptamers. All aptamers were much more stable than a random DNA strand of 55 nucleic acids, which was degraded faster than any of the aptamers (**Figure 1C**, **Supplementary Figure S2**).

Based on stability and biofilm binding, SA31 was chosen for the subsequent development of biofilm-targeting liposomes.

### 3.2 Aptamer SA31 Mediate Liposome Accumulation in *S. aureus* Biofilm

**Figure 2:**
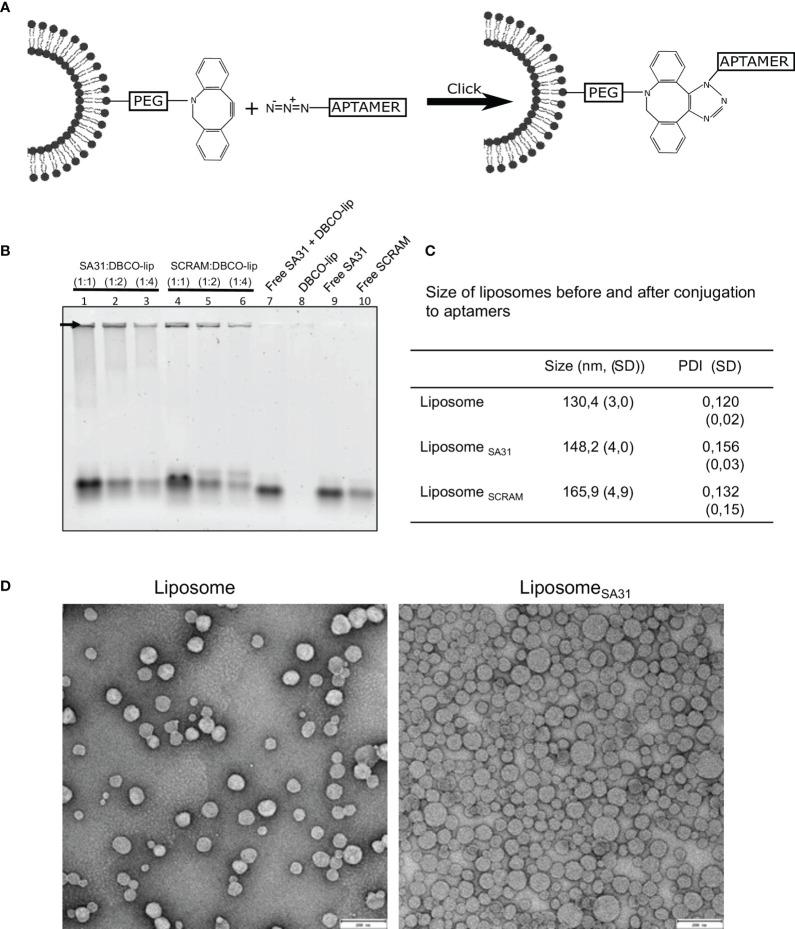
Liposome functionalization with DNA aptamers. **(A)** Scheme of liposome functionalization with DNA aptamers using N_3_-DBCO click chemistry. **(B)** Verification of liposome functionalization. Whole liposomes were loaded on the gel. Non-bound aptamers migrated into the gel lanes, while liposome-bound aptamers remained in the well (arrow). Lanes 1–6 contain aptamer-functionalized liposomes with different aptamer:lipid ratios. Lanes 7–10 contain aptamers, lipids, or both. **(C)** Characterization of liposome size by DLS before and after functionalization with aptamers (mean ± S.D., n = 3). **(D)** TEM imaging of liposomes before and after functionalization with aptamers. Scale bar 200 nm.

To investigate aptamer SA31’s capability as a targeting agent, it was conjugated to liposomes by the use of N_3_-DBCO click chemistry. In parallel, a scrambled version of aptamer SA31, named SCRAM, was also conjugated to liposomes to serve as a control for non-specific DNA-mediated interactions with the biofilm. The nucleotide composition of SA31 and SCRAM is the same, but the nucleotide sequence of SCRAM is altered, such that the tertiary structure of the folded aptamer will be different from that of SA31. Aptamers were 3′ end-modified with N_3_ and coupled to DBCO-functionalized liposomes (DBCO-lip) in various amounts (**Figure 2A**). Successful conjugation of aptamer to liposomes was confirmed by gel electrophoresis (**Figure 2B**). Aptamers conjugated to liposomes remained trapped in the well (arrow), whereas un-conjugated aptamers migrated into the lane during electrophoresis. No signal was observed in the well with DBCO lipids alone (lane 8) or if un-conjugated lipids were mixed with free aptamers (lane 7). Thus, conjugation was not due to non-specific adsorption of aptamers to liposomes.

The mean diameter of aptamer-modified liposomes showed a modest increase compared to the unmodified liposomes when measured by dynamic light scattering (DLS) (**Figure 2C**) consistent with successful conjugation of aptamers to the surface of the liposomes. Transmission electron microscopy (TEM) confirmed that unmodified and SA31-modified liposomes were similar in shape and size (**Figure 2D**).

**Figure 3:**
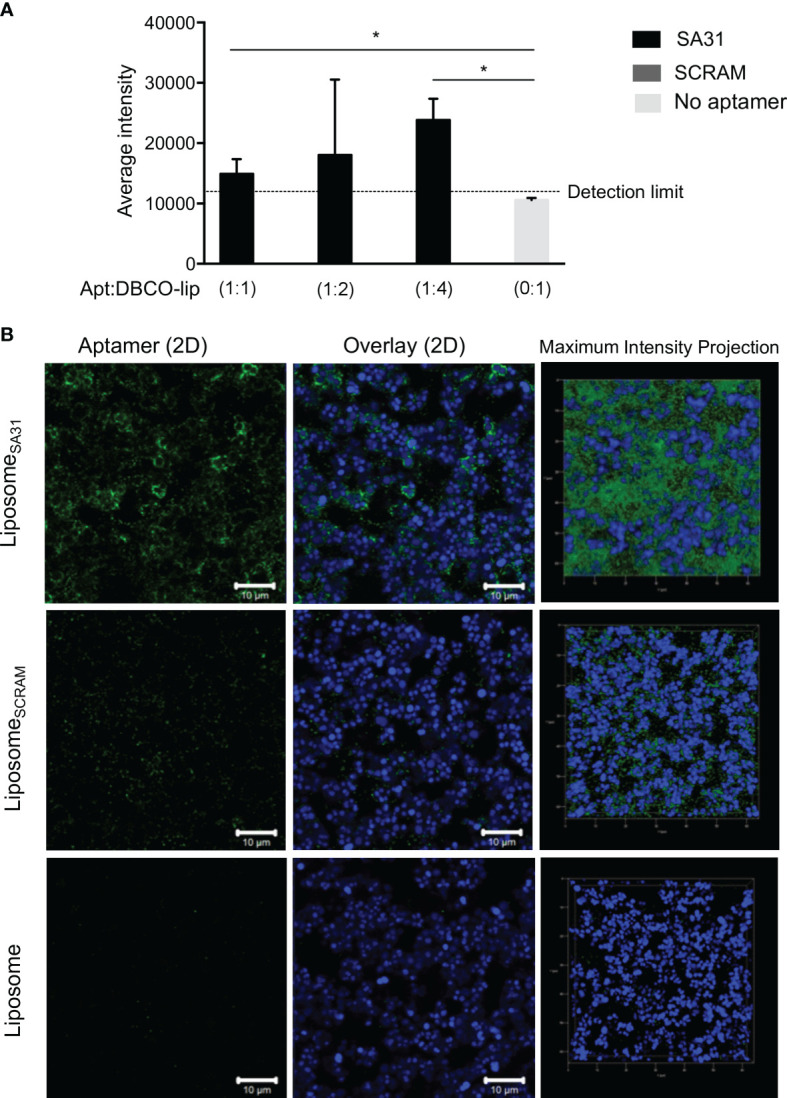
Accumulation of liposomes in *S. aureus* biofilm. *S. aureus* were grown for 24 h in BHI with 5% human plasma and incubated with liposomes for 1 h before washing off unbound liposomes. Liposomes functionalized with SA31 were compared to liposomes functionalized with SCRAM and unfunctionalized liposomes **(A)** Fluorescence intensity of sulforhodamine B-loaded liposomes retained in the biofilm (mean ± S.D., n = 3). * marks p < 0.05 (unpaired t-test). **(B)** CLSM images demonstrating accumulation of SA31-functionalized sulforhodamine B-loaded liposomes (green) (1:2 ratio between aptamer and functional DBCO groups) around *S. aureus* cells (stained with SYTO41) (blue). CLSM images were acquired with a ×100 oil immersion objective.

To demonstrate aptamer-mediated targeting of liposomes to biofilms, the liposomes were loaded with the fluorescent dye sulforhodamine B. Biofilms of *S. aureus* were incubated with the sulforhodamine B-loaded liposomes that were either unmodified or functionalized with various amounts of aptamer SA31 or SCRAM. Retention of liposomes in biofilms was quantified by using the sulforhodamine B signal in each well. Significantly higher retention of SA31-modified liposomes was observed compared to SCRAM-modified liposomes and unmodified liposomes (**Figure 3A**). Furthermore, the presence of aptamer SA31 on every fourth of DBCO groups (1:4) resulted in higher retention of liposomes in the biofilm than SA31 present on all DBCO groups (1:1) (**Figure 3A**). CLSM imaging was used to obtain detailed information on penetration, accumulation, and binding characteristics of aptamer-modified liposomes. Liposomes modified with aptamer SA31 bound to the surface of *S. aureus* and accumulated in the biofilm (**Figure 3B**). Furthermore, full penetration of the biofilm was observed. The non-specific SCRAM did not promote binding of liposomes to the surface of bacteria; however, a slight unspecific accumulation of liposomes was observed. No binding of unmodified liposomes was observed either (**Figure 3B**). Thus, aptamer SA31 is applicable as a biofilm-targeting agent on liposomes.

### 3.3 Killing Competence of Aptamer-Functionalized Antibiotic-Loaded Thermosensitive Liposomes

**Figure 4:**
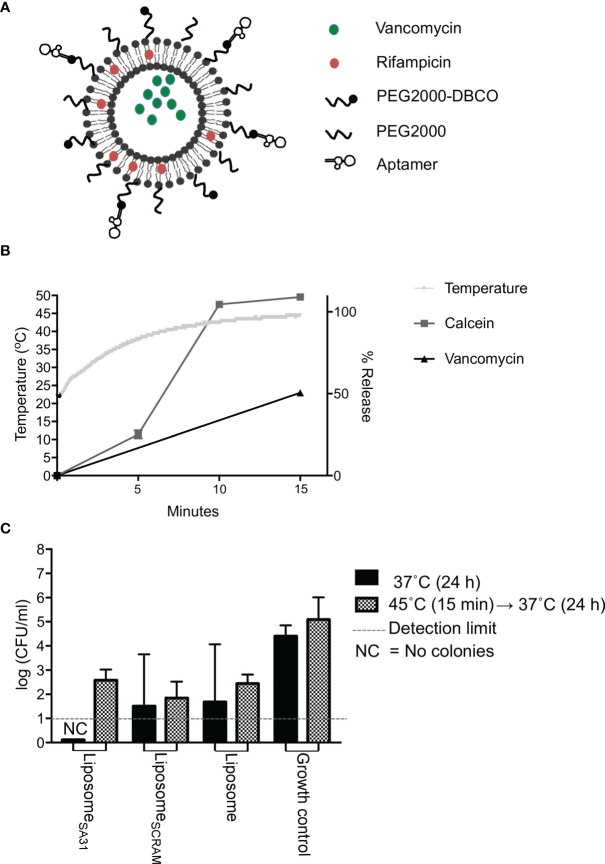
Drug release and antimicrobial effect of aptamer-functionalized antibiotic-loaded thermosensitive liposomes. **(A)** Scheme of aptamer-functionalized liposomes loaded with vancomycin and rifampicin. **(B)** Temperature change and release characteristics of calcein (model drug) and vancomycin from thermosensitive liposomes during 15 min incubation of samples in a 45°C water bath. **(C)** Antimicrobial effect of liposomes loaded with vancomycin and rifampicin. *S. aureus* biofilms were incubated with liposomes containing a total of 1,000 μg of vancomycin for 1 h at 37°C. Unbound material was removed by washing five times in PBS. Biofilms were then either incubated at 37°C immediately or placed in a 45°C water bath for 15 min first prior to a 24-h incubation at 37°C, followed by CFU enumeration. Bars show mean values, error bars = SD. * marks p < 0.05 (unpaired t-test). NC, No colony forming units were detected

Lastly, we tested aptamer SA31 as a targeting agent in a liposomal drug delivery system. In the design of this drug delivery system to *S. aureus* biofilms, the following requests were as follows: 1) the liposomes should be functionalized with a targeting agent that ensures accumulation in the biofilm, 2) a combination of drugs with synergistic effect should be included to ensure a lower concentration needed for killing, and 3) an easy release-on-demand mechanism for a burst release should be incorporated to obtain the highest possible concentration. The first request was addressed by the identification of aptamer SA31 as a biofilm-targeting agent. The drugs chosen for encapsulation were vancomycin and rifampicin (**Figure 4A**). To gauge the concentration required to eradicate the biofilm, we incubated non-targeted liposomes loaded with either vancomycin or a combination of vancomycin and rifampicin and determined the minimum biofilm eradication concentration (MBEC) value after a 24-h incubation at 37°C. Combining vancomycin with rifampicin resulted in at least a 10-fold reduction in the MBEC value for vancomycin (from 232 to 25 μg/ml, **Supplementary Table S2**). To release the antibiotics on demand, a low-temperature sensitive liposome formulation was used. We chose to heat the samples in a water bath at a temperature slightly above the melting temperature of the liposomes. In this setting, we characterized the release profile of the model drug calcein and of vancomycin. Upon 15 min in the 45°C water bath, the temperature had equilibrated and the maximum reachable release was obtained for calcein. At this time point, vancomycin release had reached 50% (**Figure 4B**). Thus, for the biofilm kill studies, we chose to heat for 15 min. We determined the MBEC value again, exposing biofilms and liposomes to 45°C for 15 min prior to the 24-h incubation at 37°C. Interestingly, the MBEC values remained the same, indicating that the antibiotics were released during the 24-h incubation period, regardless of an exposure to hyperthermia.

To evaluate the biofilm killing competence of the targeted heat-inducible drug delivery system, *S. aureus* biofilms were treated with aptamer SA31-modified antibiotic-loaded liposomes, SCRAM-modified antibiotic-loaded liposomes, or unmodified antibiotic-loaded liposomes (**Figure 4C**). Liposomes accumulated in the biofilm for 1 h before unbound material was removed by washing in PBS. Biofilms were incubated for 15 min in a 45°C water bath to trigger a bolus release from the retained liposomes. Biofilms were then incubated at 37°C for 24 h before evaluating the killing effect. As a control, treated biofilms were incubated directly at 37°C. All antibiotic-loaded liposomes killed a fraction of bacteria in the biofilms, but the heat-triggered release did not affect the antimicrobial efficacy. Targeting liposomes to the biofilm with the SA31 aptamer did not significantly increase the antimicrobial effect. However, only the SA31-modified liposomes were able to fully eradicate all viable and culturable bacteria in all biofilm samples.

## 4 Discussion

Conventional antibiotic treatment is inadequate for eradication of bacterial biofilm infections as only sublethal concentrations can be administered. In this study, we present an aptamer-targeted liposomal drug delivery system that accumulates in *S. aureus* biofilm and delivers a combination of antibiotics locally inside the biofilm in close proximity to the bacteria. This study identifies aptamers specific for *S. aureus* biofilm, and it demonstrates for the first time the potential for aptamer-mediated targeting of drug-loaded liposomes to *S. aureus* biofilm. Our study corroborates other recent studies that use aptamers to target antimicrobials to bacteria (Mao et al., 2018; Wang et al., 2018).

An obvious shortcoming is the lack of knowledge about the molecular target for the aptamer identified as a suitable targeting ligand for *S. aureus* in biofilm. This makes it difficult to predict the applicability *in vivo*. To accede to this difference and to increase our chances for a similar success *in vivo*, the *S. aureus* biofilms were grown in the presence of human plasma to enable formation of a fibrin-rich pseudocapsule and thick biofilm matrix before confirming that aptamer-functionalized liposomes could penetrate the biofilm and associate with the cell surface.

The aptamer SA31 was selected from screening nine aptamers with specificity toward either planktonic *S. aureus* cells or staphylococcal Protein A. In the screening we identified six aptamers, which bound *S. aureus* in biofilm. The molecular targets on *S. aureus* for these aptamers are unknown, but the aptamers have proven useful for detection of *S. aureus in vitro* (Wang et al., 2015), *ex vivo* (Zelada-Guillén et al., 2012; Borsa et al., 2016; Ranjbar and Shahrokhian, 2018), and *in vivo* (Santos et al., 2015; Santos et al., 2017) with specificity for type strains as well as for clinical isolates. Despite Protein A’s popularity as a molecular target on *S. aureus* for both drug delivery and detection (Suci et al., 2007; Anastasiadis et al., 2014; Meeker et al., 2016), the aptamer (PA2/8) specific for this protein did not show binding to *S. aureus* in biofilm. We ascribe this either to be due to the lack of Protein A expression on *S. aureus* in the biofilm, or a shield of the recognized epitope. The aptamers were developed in the absence of human plasma, and Protein A and matrix-binding bacterial surface proteins are likely bound by antibodies and soluble matrix proteins in human plasma present in the biofilm growth media in our experiments.

Aptamer SA31 showed good binding to *S. aureus* in biofilm (**Figure 1A**) and was investigated for its ability to mediate liposome accumulation in the biofilm. We confirmed that liposomes functionalized with SA31 did accumulate around *S. aureus* cells in biofilm and that the liposomes were able to penetrate the entire biofilm (**Figure 3B**). The binding of SA31-targeted liposomes to the surface of *S. aureus* cells was however not uniform. While liposomes surrounded some cells entirely, a neighboring cell could be completely free of liposomes. Presumably, this cell-to-cell variation in liposome binding reflects differences in expression of the molecular target. Such heterogenic expression of cell surface proteins has been observed by Brady et al. in *S. aureus* biofilm (Brady et al., 2007). In contrast to SA31, the scrambled version of the aptamer showed limited binding to bacteria in the biofilm, and unfunctionalized liposomes did not accumulate at all (**Figure 3B**). The presence of some SCRAM-functionalized liposomes may be due to unspecific interactions of the aptamer with molecules in the biofilm.

The final goal was to use the aptamer-targeted liposomes to eradicate *S. aureus* biofilm. To increase our chance of success, we encapsulated both vancomycin and rifampicin in the liposomes. This antibiotic combination has proven synergistic in several studies (Deresinski, 2009; Rose and Poppens, 2009; Niska et al., 2013; Vergidis et al., 2015; Jørgensen et al., 2016; Boudjemaa et al., 2017), and we found that with this combination we needed only one-tenth of drug-loaded liposomes for eradication of *S. aureus* biofilm (**Table S2**). We hypothesized that optimal antimicrobial effect would be obtained by releasing the content in a bolus fashion, and we therefore designed low-temperature sensitive formulation that would release the loaded antibiotics at mild hyperthermia treatment (**Figure 4B**).

Liposomes have relatively short circulation times *in vivo*, and we therefore investigated the antimicrobial effect of antibiotic-loaded liposomes that had accumulated in biofilms after only brief exposure. Liposomes were incubated with biofilms for 1 h before washing away unbound liposomes and inducing drug release by hyperthermia. While only the SA31-functionalized liposomes completely eradicated viable cells from all replicate biofilms, the result did not differ significantly from the effect of SCRAM-functionalized and non-functionalized liposomes (**Figure 4C**). Hence, antibiotics were released from liposomes in all samples, indicating either that liposomes accumulated in the biofilm regardless of targeting or that sufficient amounts of antibiotics were released from liposomes to cause cell death during the 1-h incubation prior to washing away unbound liposomes. We have evidence that some SCRAM-functionalized liposomes did accumulate in biofilms (**Figure 3B**) but found no accumulation of non-functionalized liposomes. The impact of non-functionalized liposomes on cell viability therefore suggests that some amount of antibiotics must be released before unbound liposomes are washed away.

If antibiotics are released during the first hour of incubation, it also implies that hyperthermia is not required for drug release in the biofilm. This is exactly what we observe. Either hyperthermia had no effect on the antimicrobial efficacy of liposomes (for SCRAM- or non-functionalized liposomes) or it counteracted the antimicrobial effect (for SA31-functionalized liposomes) (**Figure 4C**). Complete eradication of viable cells in any of the replicate biofilms was only observed in samples that were not exposed to hyperthermia. The antibiotics used in this study are stable at the applied temperatures (Traub and Leonhard, 1995), and adjuvant hyperthermia has previously been described to enhance the antimicrobial activity of antibiotics (Hajdu et al., 2010; Stewart et al., 2017). However, these results were obtained from long-term hyperthermia while a mild short-term hyperthermia treatment has been shown to induce cell division arrest in *S. epidermidis* (Pavlovsky et al., 2015). If the 15-min hyperthermia used in our study induces cell division arrest in *S. aureus*, it could reduce the effect of vancomycin, which targets cell wall synthesis and therefore is most effective against actively growing bacteria.

So, how were antibiotics released in the absence of hyperthermia? We speculate that lipases secreted from the *S. aureus* could degrade the liposomes, as was observed and exploited by Liu et al. (Liu et al., 2016). The presence of soluble lipases could induce drug release immediately after addition of liposomes to the biofilms and thereby cancel out the effect of hypothermia and of biofilm targeting.

In conclusion, our liposomal drug delivery system carrying a combination of synergistic antibiotics and targeted with a *S. aureus*-specific aptamer was able to eradicate *S. aureus* biofilm *in vitro*, and it presents a strategy to overcome the resistance and tolerance mechanisms of pathogenic *S. aureus* biofilms. Much remains to be optimized before such delivery systems can be implemented *in vivo.* Most importantly, drug release kinetics in the presence and absence of biofilms needs to be understood in more detail. Our data indicate that liposomes are not stable when exposed to *S. aureus* biofilms, as even non-targeted liposomes substantially reduced the number of viable cells (**Figure 4C**). If this instability is caused by the activity of lipases secreted by the bacteria, it is of great advantage for *in vivo* use, as it makes the need for hyperthermia to trigger drug release obsolete.

## Data Availability Statement

The original contributions presented in the study are included in the article/**Supplementary Material**. Further inquiries can be directed to the corresponding author.

## Author Contributions

PO, LH, BH, and HV-Q contributed to the experimental design, experimental work, and data interpretation. RM and JK proposed the project idea and contributed to the experimental planning and data interpretation in collaboration with the other authors. PO drafted the manuscript, and all authors edited the manuscript before submission. All authors contributed to the article and approved the submitted version.

## Funding

We thank the Danish Council of Independent Research (grant no. DFF 4005-00474) for funding this work.

## Conflict of Interest

The authors declare that the research was conducted in the absence of any commercial or financial relationships that could be construed as a potential conflict of interest.

## Publisher’s Note

All claims expressed in this article are solely those of the authors and do not necessarily represent those of their affiliated organizations, or those of the publisher, the editors and the reviewers. Any product that may be evaluated in this article, or claim that may be made by its manufacturer, is not guaranteed or endorsed by the publisher.
