# Supplementary material for: Aptamer-Targeted Drug Delivery for Staphylococcus aureus Biofilm
Source: Front Cell Infect Microbiol. 2022 Apr 29;12:814340. doi: 10.3389/fcimb.2022.814340 (PMC9104115; doi:10.3389/fcimb.2022.814340)
Supplement: Supplementary file 1 [file DataSheet_1.pdf]

# *Supplementary Material*

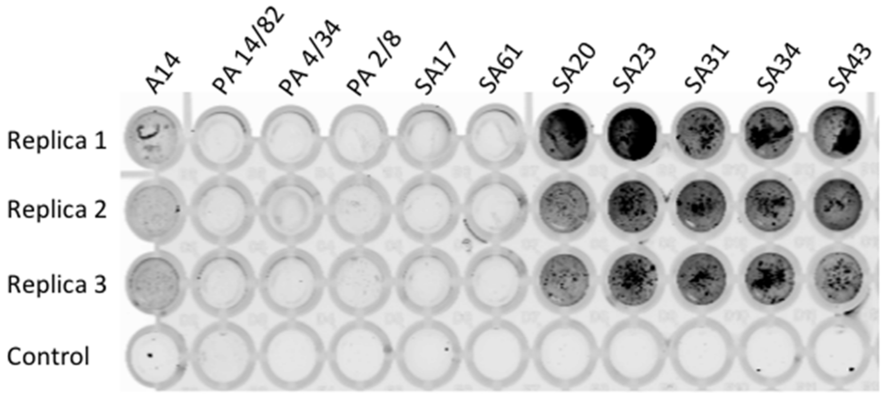


**Supplementary Figure S1** – Cy5-labeled aptamers (250 nM) screening against *S. aureus* biofilm grown in 96-well plates. After 1 h of incubation at 37 °C and three times washing, Cy5 image was taken using the Typhoon Trio Variable Mode Imager System (633 nm laser line). Each aptamer was tested on biofilm in three replicas and in an empty well as control.

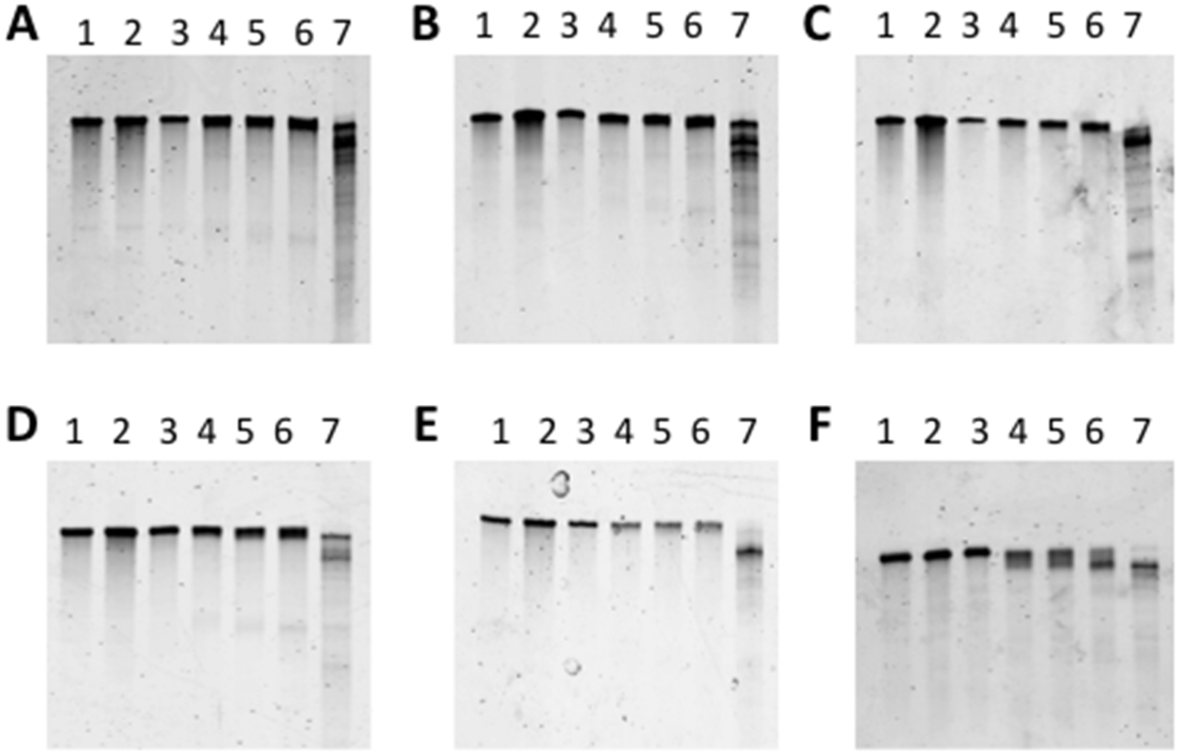


**Supplementary figure S2** – Gel images of the aptamers stability in plasma. A) SA20, B) SA23, C) SA31, D) SA34, E) SA43 and F) ssDNA control (55 nucleotides). The aptamers in buffer at 0 min and overnight in lanes 1-2, and in plasma at 0, 30, 60, 120 min and overnight (16 h) in lanes 3-7, respectively. Gel images were acquired using the Typhoon Trio Variable Mode Imager System.

Table S1: Aptamer buffer conditions and folding procedure

| Aptamer | Buffer | Folding procedure | Selected for | Reference |
|---|---|---|---|---|
| SA17<br>SA61 | 40 mM HEPES buffer pH 8.0, 5mM KCl, 1mM $CaCl_2$, 2mM $MgCl_2$, 150mM NaCl | Heat at 99°C for 2min, then gradual cooling to 37°C at a rate of 2°C / 40sec | Whole *S. aureus* cells | (Chang et al., 2013) |
| SA20<br>SA23<br>SA31<br>SA34<br>SA43 | PBS pH 7.4, 0.05% (v/v) Tween-20 | Heat at 100°C for 5min, then cool on ice for 10min | Whole *S. aureus* cells | (Cao et al., 2009) |
| A14 | PBS pH 7.4 | Heat at 95°C for 10min, then cool on ice for 10min | Whole *S. aureus* cells | (Moon et al., 2015) |
| PA2/8<br>PA4/34<br>PA14/82 | 20 mM Tris-HCl pH 7.6, 5 mM KCl, 1 mM $CaCl_2$, 10 mM $MgCl_2$, 100 mM NaCl | Heat at 90 °C for 8min, then cool at 4°C for 10min, followed by short incubation at room temperature | Protein A (PA 4/34 and 14/82 showed no binding) | (Stoltenburg et al., 2015) |

Table S2: Minimum biofilm eradication concentration (MBEC) of vancomycin alone or in combination with rifampicin in non-targeted liposomes

| | **Treatment regime** | |
|---|---|---|
| **Loaded antibiotics** | **37ºC for 24h** | **45ºC for 15 min, then 37ºC for 24h** |
| Vancomycin | >232µg/ml | >232 µg/ml |
| Vancomycin and rifampicin | 25 µg/ml | 25 µg/ml |

24h-old biofilms were grown as described in section 2.1, however in this assay biofilms were formed on peg-lids (NUNC-TSP 445497, Thermo Fisher Scientific) submerged into 96-well microtiter plates (NUNC 161093, Thermo Fisher Scientific). Liposomes containing vancomycin alone or both vancomycin and rifampicin were prepared as described in section 2.4. Their content of vancomycin was determined as described in section 2.8. Both samples of liposomes were serially diluted (two-fold in each step) in BHI. Peg-lids with 24 h old biofilms were transferred to microtiter plates with 200 µL/well of liposome and either treated for 24 h at 37 ºC or first placed in a 45 ºC water bath for 15 min to induce a burst release and then at 37 ºC for 24 h. After treatment, biofilms were rinsed from excess antibiotics by incubating the peg-lids twice for 1 min in microtiter plates with 200 µL/well PBS. The biofilms were then transferred a the recovery plate with 200 µL/well BHI and sonicated in a 45 kHz water bath sonicator for 10 min. Upon 72 h of recovery at 37 ºC, the peg-lid was discarded and the presence of planktonic growth was assessed by measuring optical density at 600 nm.

Each experiment was carried out in three biological replicates, and MBEC was determined as the concentration of antibiotic that resulted in absence of viable cells in minimum two out of the three replicates.

**References**

Cao, X., Li, S., Chen, L., Ding, H., Xu, H., Huang, Y., et al. (2009). Combining use of a panel of ssDNA aptamers in the detection of Staphylococcus aureus. *Nucleic Acids Res.* 37, 4621–4628. doi:10.1093/nar/gkp489.

Chang, Y.-C., Yang, C.-Y., Sun, R.-L., Cheng, Y.-F., Kao, W.-C., and Yang, P.-C. (2013). Rapid single cell detection of Staphylococcus aureus by aptamer-conjugated gold nanoparticles. *Sci. Rep.* 3, 1863. doi:10.1038/srep01863.

Moon, J., Kim, G., Park, S., Lim, J., and Mo, C. (2015). Comparison of Whole-Cell SELEX Methods for the Identification of Staphylococcus Aureus-Specific DNA Aptamers. *Sensors* 15, 8884–8897. doi:10.3390/s150408884.

Stoltenburg, R., Schubert, T., and Strehlitz, B. (2015). In vitro Selection and Interaction Studies of a DNA Aptamer Targeting Protein A. *PLoS One* 10, e0134403. doi:10.1371/journal.pone.0134403.
